# Association between simulated ketamine exposures and oxygen saturations in children

**DOI:** 10.4155/ipk-2022-0003

**Published:** 2023-03-07

**Authors:** Sarah Jane Commander, Daniel Gonzalez, Karan R Kumar, Tracy Spears, Michael Cohen-Wolkowiez, Kanecia O Zimmerman, Jan Hau Lee, Christoph P Hornik

**Affiliations:** 1 Duke Clinical Research Institute, Duke University, Durham, NC 27705, USA; 2 Department of Surgery, Duke University School of Medicine, Durham, NC 27705, USA; 3 Division of Pharmacotherapy & Experimental Therapeutics, UNC Eshelman School of Pharmacy, The University of North Carolina at Chapel Hill, Chapel Hill, NC 27599, USA; 4 Department of Pediatrics, Duke University School of Medicine, Durham, NC 27705, USA; 5 Department of Pediatric Subspecialities, Children’s Intensive Care Unit, KK Women’s & Children’s Hospital, Singapore

**Keywords:** ketamine, oxygen desaturation, real-world data, respiratory compromise, simulated plasma concentration

## Abstract

**Aim:**

We performed a real-world data analysis to evaluate the relationship between simulated ketamine exposures and oxygen desaturation in children.

**Materials & methods:**

A previously developed population pharmacokinetic model was used to simulate exposures and evaluate target attainment, as well as the association with oxygen desaturation in children ≤17 years treated with intravenous ketamine.

**Results:**

In 2022 children, there was no significant association between simulated plasma ketamine concentrations and oxygen saturation; however, a higher cumulative area under the curve was associated with increased odds of progression to significant desaturation (<85%), though magnitude of effect was small.

**Conclusion:**

By leveraging a population pharmacokinetic model and real-world data, we confirmed there is no relationship between simulated ketamine plasma concentration and oxygen desaturation.

Ketamine is often used in children for short, painful procedures since it achieves rapid onset of both anesthesia and analgesia through numerous mechanisms of action. Ketamine induces a state of dissociation likely due to functional and electrophysiological dissociation of the thalamo-neocortical and limbic systems [1]. Ketamine is favored for short procedures, given its limited cardiovascular effects and reduced risk of significant respiratory depression requiring mechanical ventilation [2]. Nonetheless, oxygen desaturation is a potentially significant adverse event reported in up to a third of children receiving ketamine [3]. The exact relationships between ketamine dosage, achieved systemic exposure and oxygen desaturation in children is unknown.

Drug dosing is selected to achieve systemic exposures that maximize the likelihood of a favorable response, while minimizing the risk of toxicity [4]. However, drug exposure in infants and children is often highly variable, so dose–safety relationships may be confounded and exposure assessments may be needed to identify safety signals [5]. Therefore, comprehensive pediatric safety studies should consider the impact of drug dosing, growth and maturation and disease states on exposure–safety relationships [6].

Using drug concentration measurements from biospecimens to characterize exposure in large-scale, pediatric safety studies is challenging and impractical [7]. Population pharmacokinetic (PopPK) models created from small studies in similar patient populations present an alternative and can be used to predict individual drug exposures in new patients by assessing the effects of dosing, subject characteristics and the extent of interindividual (IIV) and intraindividual variability [8]. When applied to dosing and clinical real-world data (RWD) extracted from electronic health records (EHR), PopPK model simulations can evaluate associations with efficacy and safety data recorded in the EHR. RWD extracted from EHR is a promising source of real-world evidence to augment pediatric safety data and is increasingly accepted by the US FDA [1]. Applying PopPK model-derived exposure simulations significantly enhances the value of EHR RWD in the assessment of exposure–safety relationships for pediatric drugs. We applied this method to ketamine, which is rarely associated with clinically significant oxygen desaturation, but whose exposure–safety relationship in children is largely unknown [1].

## Materials & methods

### Data source & formatting

From a previously developed multicenter EHR informed RWD registry [9], we identified all children ≤17 years of age treated with intravenous infusion ketamine. The repository was created from nine sites around the USA with 386,159 inpatient encounters from 264,709 children using the Patient Centered Clinical Research Network data model. Demographic data and extracorporeal membrane oxygenation (ECMO) support (using code number 33964) at the time of ketamine dosing were extracted. Only one encounter per child was included. For each included drug dose, we captured drug amount, date, time and duration of infusion. Children with any missing demographics were excluded. No assumptions were made from missing information in the chart and no clinical data were imputed.

We collected baseline oxygen support and all oxygen saturation measurements (%), for up to 24 h after the first administered ketamine dose. We defined the observation period as the time from ketamine dose administration until the last recorded oxygen saturation or 24 h, whichever came first. Dates and times of measurement of oxygen saturation recorded in the EHR were used as the time points for exposure simulation. Oxygen saturation cutoffs of 92% and 85% were chosen to represent mild and significant desaturation, respectively [10,11]. Changes from baseline values before drug exposure were calculated at each assessment time point. We defined baseline as the last saturation measurement available prior to ketamine administration.

### Exposure simulation

We used a previously developed ketamine pediatric PopPK model to simulate exposures in the children identified from the RWD registry [12]. The patient population extracted from the EHR utilized a similar inclusion criterion as that included in the PopPK model: children ≤17 years of age who received at least one dose of intravenous ketamine. The model was created from intravenous and intramuscular doses of ketamine from two multicenter trials. The PopPK model was previously developed by opportunistic PK plasma sampling and the equations used for simulation are as follows:

$$CL\;\left(\frac{L}{h}\right)=38.9\times {\left(WT\;(kg)/\;70\right)}^{0.75}\times \;2.35^{ECMO}$$

$$V_{c}\;\left(L\right)=32.8\times (WT\;(kg)/\;70)$$

$$Q\left(\frac{L}{h}\right)=54.9\times {\left(WT\;(kg)/\;70\right)}^{0.75}$$

$$V_{p}\;\left(L\right)=152\times (WT\;(kg)/\;70)$$

Where CL is systemic clearance, WT is weight, ECMO support (ECMO = 1) or absence (ECMO = 0), V_c_ is the central compartment volume of distribution, Q is intercompartmental clearance and V_p_ is the peripheral compartment volume of distribution. While intravenous and intramuscular doses were used to develop this model, and intramuscular bioavailability estimated, we only simulated exposures after intravenous administration in this RWD analysis. For the simulation, fixed- and random-effect parameters were fixed at the final model estimates. Individual predictions of ketamine concentrations at each time point of oxygen saturation assessment were chosen to represent drug exposure, as these simulated values incorporated both the effects of clinical characteristics included as covariates in the PopPK model and remaining IIV. The original model development [12] reported precision of model-based simulations using repeated simulations of individual concentration time profiles. This was not repeated in this analysis of simulated exposure–response relationships in individual participants in this model.

The dose of intravenous ketamine administered and the duration of infusion were extracted from the RWD registry. Any intravenous doses of ketamine administered within 24 h of the first intravenous dose of ketamine were included in the simulations, but intravenous ketamine administered >24 h after the first dose were considered a separate ketamine treatment episode and not included in this analysis. The timing of plasma concentration simulations is the time after the first dose during which oxygen saturations were documented in the RWD registry up to 24 h after the start of the first intravenous dose of ketamine. This was chosen, since the targeted objective of this simulation is to evaluate relationships between oxygen saturations and simulated ketamine plasma concentrations, and not to comprehensively characterize the plasma concentration time profiles of ketamine following all dosing simulations.

A sensitivity analysis was also performed wherein population predictions of ketamine concentration, excluding IIV, were used, with all other simulation parameters kept identical. All exposure simulations were performed using the Nonlinear Mixed-Effect Modeling software (NONMEM^®^ version 7.4.3, Icon Development Solutions, MD, USA) with run management performed using Pirana (version 2.3.8) [13]. Area under the concentration-time curve (AUC_0-inf_) targets for safety have been previously reported for ketamine in adults following a single dose (AUC_0-inf_ 3000 ng*h/ml in 12 critically ill adults with brain or spinal cord injury) [2]. Consequently, we also calculated the cumulative area under the curve (AUC) in NONMEM at each time point for the subject’s exposure to ketamine according to the equation:

$${AUC}_{cum}=\;\int_{0}^{T}C_{p}dt.$$

where *C_p_* is the plasma concentration of ketamine and *dt* represents change over time from ketamine administration to each assessment time point. Previously published plasma ketamine plasma concentration cutoffs were used to assess therapeutic success: 100 ng/ml corresponding to analgesic effect, 750 ng/ml corresponding to awakening from anesthesia, 1000 ng/ml corresponding to arousal with verbal stimulus and 1500 ng/ml corresponding to arousal with painful stimulus [14–16]. Plasma concentrations and cumulative AUCs were simulated and calculated at each saturation measurement time point after ketamine administration up to a maximum of 24 h post administration.

### Statistical analysis

We used count (with percentages) and medians (with 25th and 75th percentiles) to describe categorical and continuous variables, respectively. We stratified and compared the distribution of study variables by age, time after dose and clinically relevant variables, including level of respiratory support at the time of drug administration using the Chi-square, Fisher’s exact or Wilcoxon rank-sum test, where appropriate. We performed multivariable logistic regression to evaluate subject–level relationships between exposure measurements (maximum ketamine concentration and cumulative AUC) and oxygen shift from baseline to significant desaturation (<85%) at any time during the observation period, adjusting for subject postnatal age (PNA) and receipt of mechanical ventilation. We report odds ratios (ORs) with 95% CIs. We defined statistical significance as a p-value < 0.05. We performed all statistical analyses using Stata 15.1 (TX, USA). This study was approved by the Duke Institutional Review Board with a waiver of informed consent.

## Results

We included 2022 children with a medianPNA of 3.1 years (interquartile value [IQV]: 0.8–8.9) who received 4904 intravenous doses of ketamine at a median dose of 1 mg/kg (IQV: 0.5–1.2) (Table 1). These demographic variables were very similar to those from the previously created PopPK model, including median age, weight and gender distributions [12]. The median value of the first simulated individual plasma concentration of ketamine was 290.3 ng/ml (IQV: 0–838.6) at a median of 0.08 h (IQV: 0.03–0.25) post ketamine administration. The median value of the highest plasma concentration of ketamine achieved in all children was 1489.8 ng/ml (IQV: 675.9–2794.1) at a median of 0.25 h (IQV: 0.17–0.6). The median-simulated plasma concentration of ketamine was 218.2 ng/ml (IQV: 69.3–682.3) at a median of 2.3 h (IQV: 0.5–5.6) post first dose. The median cumulative AUC over the entire observation period was 1800 ng*h/ml (IQV: 1000–3300). ECMO support was provided to 25/2022 (1%) children at the time of ketamine administration. The number of children who achieved one plasma concentration of at least 100 ng/ml, 750 ng/ml, 1000 ng/ml and 1500 ng/ml was 1965 (97%), 1464 (72%), 1287 (64%) and 1010 (50%), respectively.

**Table 1.** Extracted clinical data of children exposed to ketamine.

Clinical data extracted	n = 2022
Postnatal age (years)	3.1 (0.8–8.9)
Weight (kg)	14 (8–28)
Female sex	904 (45%)
Supported with ECMO	25 (1%)
Ketamine dosing (mg/kg)	1 (0.5–1.12)
Number of doses per subject	2 (1–3)
Simulated plasma concentration (ng/ml)	218.2 (69.3–682.3)
Simulated cumulative plasma AUC (ng*h/ml)	1800 (1000–3300)
Baseline oxygen saturation	98 (89–100)
Baseline respiratory support: None/room air Nasal cannula, face mask, tent or oxyhood High-flow nasal cannula Other noninvasive ventilation Invasive mechanical ventilation	1859 (92%) 38 (2%) 10 (<1%) 37 (2%) 78 (4%)
Saturation shift from normal baseline: Remain normal To mild desaturation To significant desaturation	1221 (61%) 53 (3%) 139 (7%)

Data shown are median (range) or counts (%).

AUC: Area under the curve; ECMO: Extracorporeal membrane oxygenation.

Median baseline oxygen saturation was 98% (IQV: 89–100). At the time of ketamine administration, 450 (22%) children had an oxygen saturation less than 85%. Only 163 (8%) children were receiving noninvasive or invasive respiratory support at the time of ketamine administration. The median of the lowest recorded saturation for each subject over the observation period was 93% (IQV: 88–96), and 339 (17%) children had at least one recorded saturation less than 85%.

**Figure 1.**
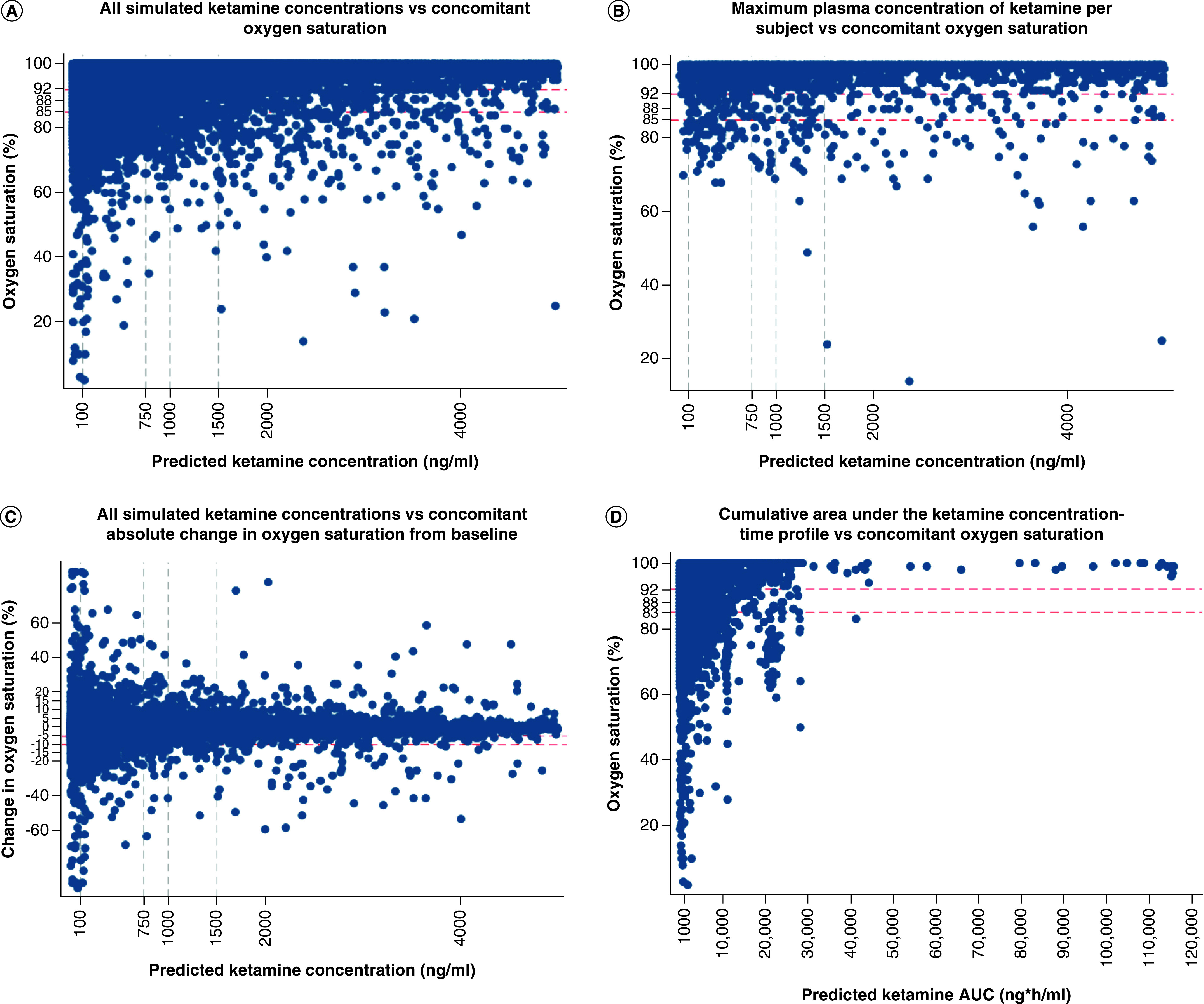
Relationship between simulated plasma ketamine exposure and oxygen saturation. Relationship between simulated plasma ketamine exposure and oxygen saturation according to: **(A)** all simulated ketamine concentrations versus concomitant oxygen saturation; **(B)** maximum plasma concentration of ketamine per subject versus concomitant oxygen saturation; **(C)** all simulated ketamine concentrations versus concomitant absolute change in oxygen saturation from baseline; and **(D)** cumulative area under the ketamine concentration-time profile versus concomitant oxygen saturation. Oxygen saturation cutoff values of 92, 88 and 85% are depicted with horizontal red dashed lines. Ketamine plasma concentrations cutoffs previously associated with efficacy are shown with horizontal grey dashed lines.

**Figure 2.**
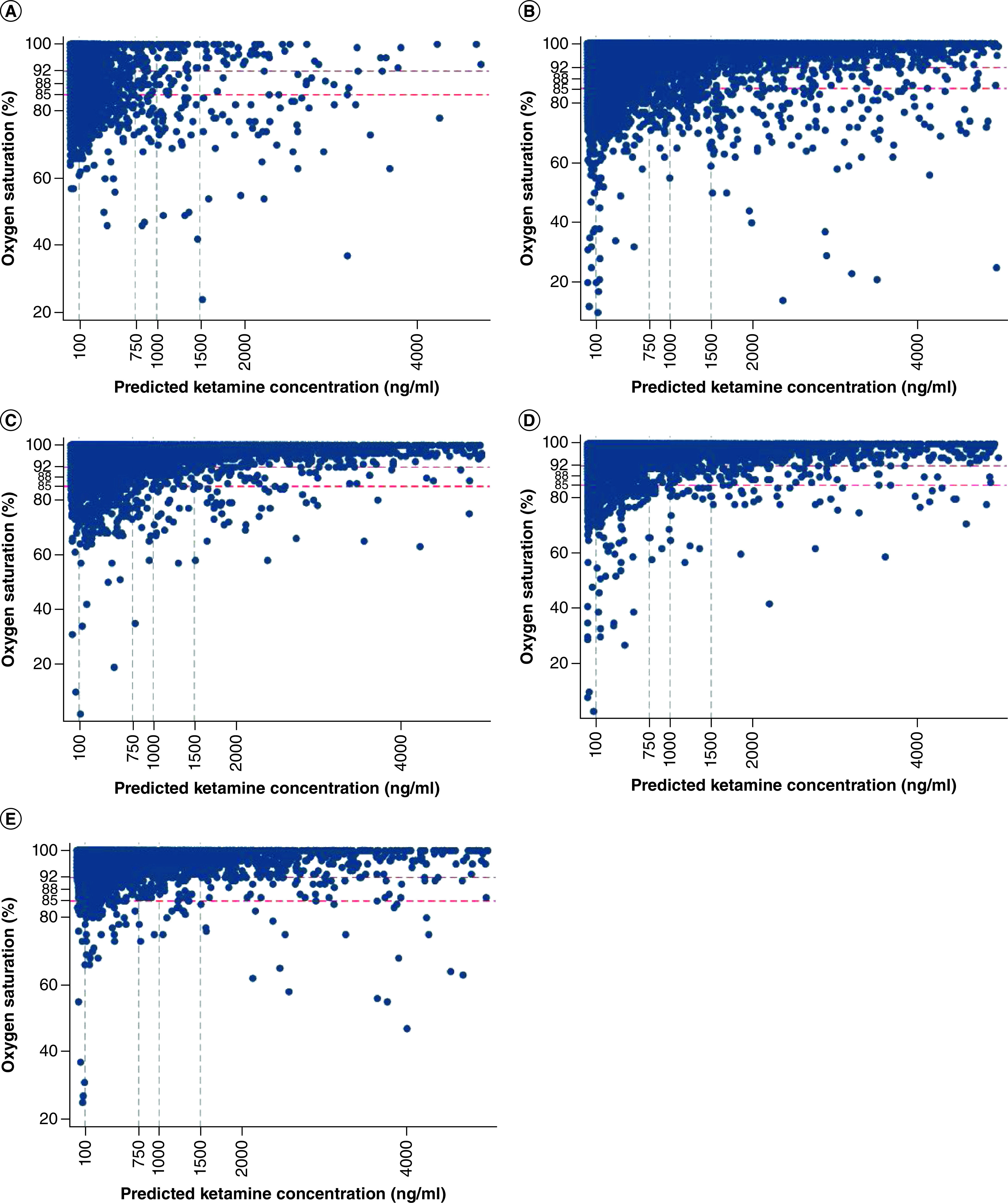
Relationship between simulated ketamine exposure and systemic oxygen saturations by postnatal age. Relationship between simulated ketamine exposure and systemic oxygen saturations by postnatal age according to: **(A)** ≤1 month of age; **(B)** >1 month to 2 years of age; **(C)** >2 years to 6 years of age; **(D)** >6 years to 12 years of age; and **(E)** >12 years of age. Oxygen saturation cutoff values of 92, 88 and 85% are depicted with horizontal red dashed lines. Ketamine plasma concentrations cutoffs previously associated with efficacy are shown with vertical grey dashed lines.

**Figure 3.**
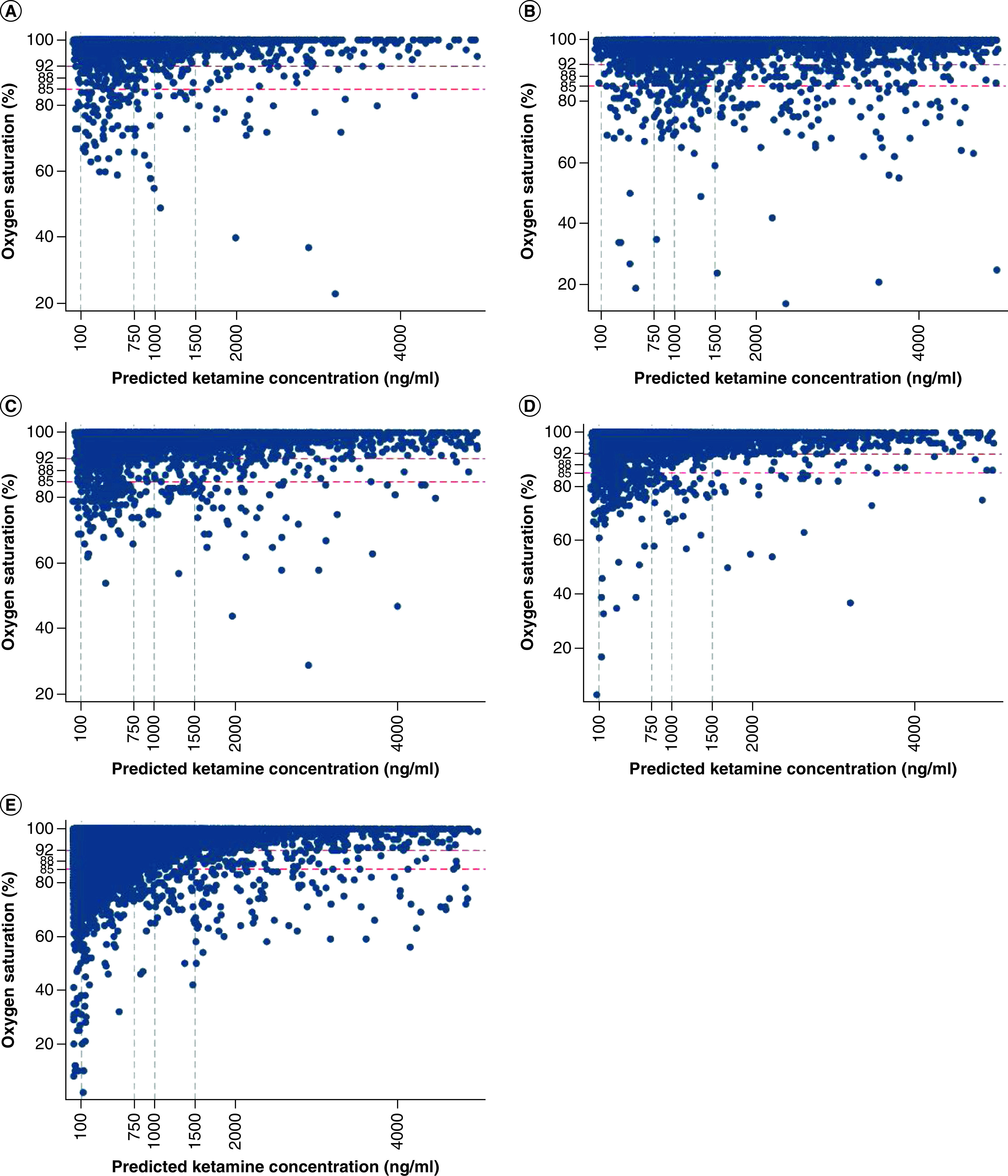
Relationship between simulated ketamine exposure and systemic oxygen saturations stratified by time after first dose. Relationship between simulated ketamine exposure and systemic oxygen saturations stratified by time after first dose according to: **(A)** ≤5 min after first dose; **(B)** >5–15 min after first dose; **(C)** >15–30 min after first dose; **(D)** >30–60 min after first dose; and **(E)** >60 min after first dose. Oxygen saturation cutoff values of 92, 88 and 85% are depicted with horizontal red dashed lines. Ketamine plasma concentrations cutoffs previously associated with efficacy are shown with vertical grey dashed lines.

Scatter plots did not reveal a significant association between simulated population plasma ketamine concentrations and oxygen saturations (Figure 1), including plotting the oxygen saturation at the time of maximum simulated plasma ketamine concentration, evaluating changes from baseline oxygen saturation and plotting the simulated cumulative AUC. Plots were similar when stratified by age (Figure 2) and time post dose (Figure 3). Results were similar when plotting simulated population predictions instead of individual predictions (Data not shown).

We observed higher simulated maximum plasma concentrations of ketamine in children with normal baseline saturation (>92%) who had at least one documented saturation of less than 85% (median 1831 ng/ml [IQV: 836–3,249]) compared with those who maintained normal saturations (1481 ng/ml [IQV: 694–2,747], p = 0.03). Nevertheless, this association did not retain statistical significance in a multivariable analysis controlling for PNA and baseline level of respiratory support (Table 2). Instead, in adjusted analysis, higher simulated cumulative AUC was associated with increased odds of progression to significant desaturation (<85%), though the magnitude of the effect was small (7% higher odds with increasing cumulative AUC).

**Table 2.** Adjusted association between the highest simulated plasma ketamine concentration or the simulated cumulative area under the concentration versus time curve and drop in oxygen saturation from normal at baseline to <85% during the observational period, controlling for postnatal age and baseline level of respiratory support.

Ketamine exposures	OR (95% CI)
Maximum plasma ketamine concentration (ng/ml)	1.00 (0.99–1.01)
Cumulative AUC over the study period (ng*h/ml)	1.07 (1.02–1.12)

AUC: Area under curve; OR: Odds ratio.

## Discussion

We successfully linked predicted ketamine exposures based on standard of care intravenous ketamine doses and clinical characteristics with oxygen saturation measurements documented in the EHR. Our simulated ketamine concentrations were within the range and variability of values previously reported in children [12]. While we did not find a statistically significant relationship between predicted exposures for ketamine and occurrence of desaturation, our results support the feasibility of our proposed approach: develop a PopPK model using opportunistically collected PK data and apply it to routinely collected RWD captured in the EHR to study exposure–safety relationships.

EHR data have been used to characterize dose–safety relationships of drugs in children, but exposure assessments remain limited predominantly to drugs undergoing routine therapeutic drug monitoring [17]. Methods that permit quantification of exposure for all drugs would greatly enhance the value of EHR RWD for pediatric drug safety assessments. Our cost-effective and efficient approach leverages PopPK models, which can be developed in relatively small studies, to simulate exposures in larger pediatric populations [18]. This approach enhances the values of RWD from the EHR to generate real-world evidence of pediatric drug safety assessments, where relationships may depend on drug exposures that vary with child maturation and disease processes.

Respiratory decompensation resulting in systemic desaturation, primarily because of upper airway compromise, is the most common pediatric adverse event associated with ketamine administration, reported in 13–33% of children. We observed a similar prevalence of significant desaturation (<85% on pulse oximetry) in our cohort, with 17% of children affected. In our study, there was no relationship demonstrated between exposure and desaturation; however, since desaturation is believed to occur primarily because of loss of upper airway control and ability to clear oral secretions, it is possible that concentrations associated with anesthetic effects of ketamine (>1500 ng/ml) would also be associated with this unwanted adverse event [16]. The lack of relationship between ketamine exposure and desaturation may also be because no children in our simulation achieved concentrations >1500 ng/ml. In a study by Grunwell *et al.* of 22,645 children who underwent ketamine sedation, desaturation occurred in nearly 2% of their cohort and was the most significant adverse event; exposure was not evaluated [19].

Prior prospective pediatric studies assessing ketamine exposures and safety have been limited by small sample sizes, with typically fewer than 50 children enrolled per trial and limited exposure data. Our novel approach resulted in a greater sample size than all prior prospective trials of ketamine combined [1,20]. Despite this advantage, we were still unable to find an association between systemic ketamine exposure and desaturation. We speculate that interventions known to prevent desaturation associated with upper airway obstruction were likely performed, but not captured, in the EHR. Furthermore, we were limited to reviewing associations between simulated exposures and the oxygen saturations documented in the EHR, which may not capture unrecorded desaturations.

Alternatively, CNS exposures, rather than plasma, may correlate with changes in innervation of the muscles controlling the upper airway and the salivary glands producing airway secretions, both of which may ultimately lead to lack of airway control, laryngospasm and desaturation [1]. A study in mice found that there is a linear correlation of plasma to cerebral spinal fluid concentrations of ketamine, which would suggest that there is rapid dissociation between these compartments [21]. Studies in humans are necessary to confirm this finding, as well as studies in neonatal and pediatric populations, to determine if cerebral spinal fluid concentrations can be inferred from plasma concentrations. Modern magnetic resonance imaging techniques have confirmed that ketamine plasma concentrations around 200 ng/ml are associated with changes in thalamic and suprathalamic function, which may lead to loss of airway control and subsequent desaturation [1]. Studies evaluating the relationship between CNS exposures and safety are challenging to conduct and have not been reported to date, but opportunities may exist when cerebral spinal fluid sampling procedures are being performed during which ketamine is administered for analgesia or sedation.

Almost all children achieved simulated exposure levels consistent with analgesic effect (97%); however, fewer achieved anesthetic effects including spontaneous wakening from anesthesia (72%), waking with arousal to verbal stimulus (64%), and only 50% achieved anesthesia that would prevent arousal with painful stimulus. Though we are unable to extract indication for ketamine, this finding does suggest that most children would retain a level of wakefulness after administration that may preclude safe performance of more invasive and painful procedures that require anesthetic levels of sedation. Notably, another report has commented on the lower doses of ketamine used in routine clinical care today as compared with doses originally tested in adults and children [22]. Alternatively, this simulation may not capture the true maximum exposure, due to ketamine’s fast mechanism of action. The median value of the first simulated plasma concentration of ketamine was 290.3 ng/ml and simulated at a median of 0.08 h (0.03–0.25) after ketamine administration. As compared with the original paper describing the PopPK model, the time to maximum dose is shorter in this report and the concentrations are slightly lower [12], perhaps because only 1 mg/kg dose was used in this simulation, whereas larger doses (up to 10 mg/kg) were used in the PopPK model development paper. The reported maximums in this report do mirror maximum concentrations in the literature following 1 and 1.5 mg/kg iv. doses in excess of 2500 and 5000 ng/ml. Since ketamine has maximal effect within minutes, higher concentrations associated with oxygen desaturation may have been missed in our simulation [1].

## Limitations

Our study had some limitations. First, we only examined single doses of administered ketamine; therefore, cumulative AUC was used to examine ketamine exposure at the time of saturation measurement since this was often after the duration of ketamine infusion, rather than AUC_0-inf_ or AUC–time curve over the dosing interval (AUC_0-tau_). Second, there was a very limited number of children on ECMO support at the time of ketamine administration, so future studies focusing on these children should be performed. Third, current methods of extracting data from the EHRs are mostly restricted to objective data points captured in individual fields. We performed an analysis to capture a potential safety event of clinically significant hypoxemia; more complex safety events (e.g., emergence delirium, which occurs in approximately 5% of children, but up to 30% of adults after ketamine therapy) [23], would require additional analyses, potentially facilitated through natural language processing, creating a reproducible computable phenotype for complex conditions of interest, and other advanced artificial intelligence strategies in the near future [24].

Fourth, while exposure simulation is an attractive approach, it is certainly not as robust as actual concentration measurements and relies on the assumption that the developed PopPK model will adequately characterize the drug characteristics in the simulated population. In particular, the significant IIV that remained in the PopPK model used for exposure simulations could mask actual relationships between exposures and safety events by introducing excessive variability in the simulations. To address this, we repeated predicted exposure–safety relationship assessment using population predictions, which did not differ from the findings shown here. Furthermore, other covariates that were not significant in the model development or were not recorded in the studies that informed the model development (e.g., liver function or exposure to certain concomitant medications) may significantly alter exposures in individual participants. We also only simulated iv. dose and did not predict any other route of administration for model simplicity and to avoid additional variability. Finally, the EHR approach is constrained by the timing of dosing administration and safety assessments performed per standard of care. This may further limit its ability to characterize safety profiles, and introduce potential errors into the simulations, due to imprecisely recorded dose amounts or times relative to safety assessments. Simulations using PK/PD models, or semi-physiologic models that can predict tissue exposures (e.g., physiologically-based PK modeling) may be able to better characterize the exposure–response relationships, including insufficiently comprehensive RWD, but are beyond the scope of our analysis.

## Conclusion

We successfully leveraged a PopPK model and EHR data to confirm the absence of a relationship between simulated plasma concentration of ketamine and oxygen desaturation. These findings are consistent with smaller prospectively collected data from prior studies and support the broader applicability of our approach to generate real-world evidence on pediatric drug exposure–safety relationships.

**Summary points**

- We evaluated a real-world data (RWD) driven approach to study the relationship between plasma concentrations of ketamine and oxygen desaturation in children.
- We used a multi-institutional RWD registry to extract baseline oxygen support, intravenous ketamine doses and oxygen saturations obtained per routine clinical care for up to 24 h after ketamine administration.
- Using a previously developed pediatric population pharmacokinetic model of ketamine, we simulated ketamine plasma concentrations and evaluated target attainment, as well as the association with oxygen desaturation in children ≤17 years treated with intravenous ketamine.
- In 2022 children, there was no significant association between simulated plasma ketamine concentrations and oxygen saturation; however, a higher cumulative area under the curve was associated with increased odds of progression to significant desaturation (<85%), though magnitude of effect was small.
- By leveraging a population pharmacokinetic model and RWD, we were able to characterize the relationship between simulated exposures and desaturation in a large cohort of children.
